# Affinity matters for IgE‐blocking activity of allergen‐specific antibodies

**DOI:** 10.1111/all.15746

**Published:** 2023-04-21

**Authors:** Maria R. Strobl, Hilal Demir, Gerhard Stadlmayr, Florian Stracke, Robert Hoelzl, Barbara Bohle, Gordana Wozniak‐Knopp

**Affiliations:** ^1^ Institute of Pathophysiology and Allergy Research, Center for Pathophysiology, Infectiology and Immunology Medical University of Vienna Vienna Austria; ^2^ Department of Biotechnology, Institute of Molecular Biotechnology University of Natural Resources and Life Sciences (BOKU) Vienna Austria

To the Editor,

It is well recognized that allergen immunotherapy (AIT) triggers the production of IgE‐blocking IgG and IgA antibodies (Abs) that prevent allergen‐induced IgE‐mediated effector cell activation.^1^ Recently, passive immunotherapy with IgE‐blocking human monoclonal Abs (mAbs) specific for the major allergens Fel d 1 (cat) or Bet v 1 (birch pollen) significantly reduced the respiratory symptoms of cat‐ and birch pollen‐allergic individuals, respectively.^2^, ^3^ These studies involved cocktails comprising two to three mAbs recognizing diverse epitopes on either allergen with subnanomolar affinity.^2^, ^4^ Notably, the cocktail of two Fel d 1‐specific mAbs displayed stronger inhibitory activity than a similar concentration of polyclonal Fel d 1‐specific IgG Abs purified from individuals after clinically effective cat‐AIT.^2^ The latter bound Fel d 1 with an apparent affinity ranging from 2.7 to 3.9 nM. These observations suggested a connection of IgE‐blocking bioactivity and affinity. However, this correlation was not yet experimentally demonstrated.

We previously reported that the reduction of apple‐induced symptoms after 16‐week sublingual immunotherapy with recombinant (r) Mal d 1 correlated with the induction of IgE‐blocking IgG1 Abs.^5^ Here, peripheral blood mononuclear cells from a successfully treated individual as documented by reduced allergic reactions in open food challenges who displayed Mal d 1‐specific IgE‐blocking IgG1 Abs served as source for engineering of specific mAbs by the Fab yeast display library technology (see Online Supplement). Eight monoclonal IgG1 Abs could be expressed at a yield of >20 mg/L and displayed monomeric profiles in size exclusion chromatography (SEC) (Figure S2A). The mAbs K1.1, K1.2, K2.4, and K2.9 showed strong binding to rMal d 1 in ELISA (Figure S2B). Their IgE‐blocking activity was tested in basophil inhibition assays (see Online Supplement). mAbs were added to rMal d 1 at molar ratios of 1:1, 10:1, and 100:1, prior to addition to heparinized blood from untreated apple‐allergic individuals collected after informed consent and ethical clearance by the local ethics committee (EK1344/2018). The murine mAbs BIP1 (Mal d 1‐binder) and BIP3 (non‐Mal d 1‐binder) served as controls. Mean values of inhibition for each mAb were calculated per donor and summarized in Figure 1A. None of the mAbs reduced allergen‐induced basophil activation at a 1:1 ratio. At 10‐fold molar excess BIP1 displayed a stronger inhibitory activity than BIP3, reaching significant difference at a 100‐fold excess. K1.1, K1.2, and K2.4 showed weak inhibitory activity at 10:1 which increased at 100:1. K2.9 enhanced basophil activation (data not shown) and was therefore excluded.

**FIGURE 1:**
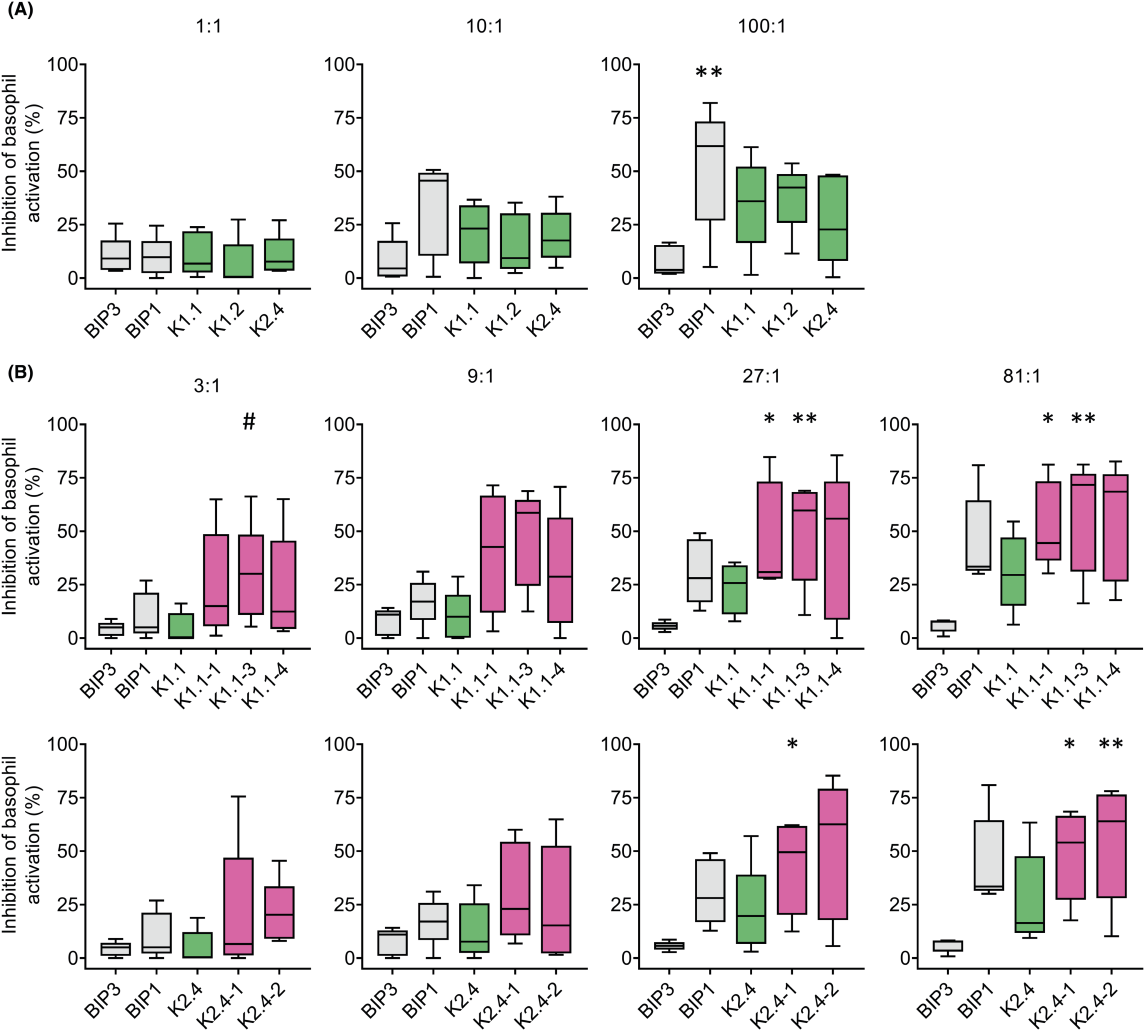
IgE‐blocking activity of Mal d 1‐specific mAbs. mAbs were incubated with titrated amounts of rMal d 1 at indicated molar ratios prior to addition to heparinized blood from apple‐allergic donors. BIP3 and BIP1 served as negative and positive control, respectively. CD63 expression on CD123^+^CCR3^+^ cells was assessed by flow cytometry. For each donor, all assays with rMal d 1 concentrations inducing 18%–69% CD63^+^ basophils were normalized to 100% activation and the percentage of inhibition by each mAb was calculated. Mean values for each mAb were calculated per donor and summarized in boxplots with Tukey whiskers. (A) Blocking activity of K1.1, K1.2, and K2.4 on basophils from five donors. (B) Comparison of K1.1 and K2.4 parental and affinity‐improved descendant mAbs (*n* = 5); Friedman test and Dunn's post hoc test; *P ≤ .05, **P ≤ .01 for the difference to BIP3, ^#^ *P* ≤ .05 for the difference to parental mAb.

K1.1 and K2.4 were selected for affinity improvement by light chain pool expansion (all methods are described in the Online Supplement). Altogether, this approach resulted in five descendant mAbs with excellent expression characteristics, monodisperse SEC profiles (Figure 2A), and stronger rMal d 1‐binding than the parental mAb in ELISA (Figure 2B). The amino acid sequences of the complementarity‐determining regions (CDR) 3 of the light chains of the different descendants of K1.1 and K2.4 showed a strong consensus (Figure 2C). Surface plasmon resonance confirmed a twofold to fivefold enhanced affinity of the descendant mAb (Figures 2B and S3). In basophil inhibition assays, all descendants showed stronger IgE‐blocking activity than the respective parental mAb that increased with the molar excess to allergen (Figure 1B). As expected from the polyclonal IgE‐response of allergic individuals, none of the single mAbs reached 100% of inhibition. Still, their inhibitory bioactivity corresponded to their affinity (Figure 2D). Here, we employed allergen‐specific IgG1 Abs as they dominate IgE‐blocking in the early phase of AIT.^5^, ^6^ Nevertheless, the correlation of affinity and inhibitory bioactivity also applies to IgG4 because previous work has shown that the isotype is not decisive for IgE‐blocking.^7^

**FIGURE 2:**
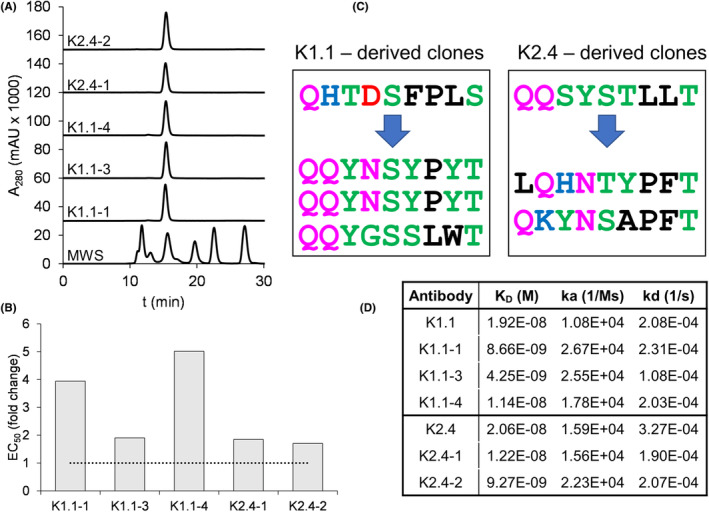
Characterization of descendant mAb. (A) Size exclusion profiles; MWS, molecular weight standard. (B) Fold change of EC_50_ values of rMal d 1‐binding in relation to parental mAbs normalized to 1. (C) Amino acid sequences (black: hydrophobic, green: polar, purple: neutral, blue: basic, red: acidic) of light chain CDR3 of parental (top) and descendant mAb. (D) Binding kinetics and affinity to rMal d 1 assessed by surface plasmon resonance; K_D_, (equilibrium) dissociation constant; k_a_, association rate constant; k_d_, dissociation rate constant.

In summary, this study demonstrates that IgE‐blocking bioactivity enhances with both amount and affinity of allergen‐specific Ab. Hence, allergen‐specific mAbs of highest affinity should be favored for optimal success of passive immunotherapy of allergy.

## CONFLICT OF INTEREST STATEMENT

BB reports grants from the Austrian Science Funds, Austrian Jubiläumsfonds, Danube Allergy Research Cluster, Country of Lower Austria, and Medical University of Vienna, Austria, during the conduct of the study and personal fees from AllergenOnline outside the submitted work. GWK reports grants from Christian Doppler Society and Austrian Science Funds outside the submitted work. The other authors have nothing to declare.

## Supporting information


Appendix S1.
