# Supplementary material for: Affinity matters for IgE‐blocking activity of allergen‐specific antibodies
Source: Allergy. 2023 Apr 21;78(9):2543–6. doi: 10.1111/all.15746 (PMC10952977; doi:10.1111/all.15746)

**Online Supplementary**

**Affinity matters for IgE-blocking activity of allergen-specific antibodies**

Maria R. Strobl*^1^, Hilal Demir*^1^, Gerhard Stadlmayr^2^, Florian Stracke^2^, Robert Hoelzl^2^, Barbara Bohle*^1^, Gordana Wozniak-Knopp*^2^

^🞹^These authors contributed equally to this work.

^1^Institute of Pathophysiology and Allergy Research, Center for Pathophysiology, Infectiology and Immunology, Medical University of Vienna, Vienna, Austria

^2^Institute of Molecular Biotechnology, Department of Biotechnology, University of Natural Resources and Life Sciences (BOKU), Vienna, Austria

**Corresponding author**

Barbara Bohle, PhD

Institute of Pathophysiology and Allergy Research, Center for Pathophysiology, Infectiology and Immunology, Medical University of Vienna, Austria,

Waehringer Guertel 18-20, 1090 Vienna, Austria

Phone: 0043-1-40400-51140, Fax: 0043-1-40400-61880

[barbara.bohle@meduniwien.ac.at](mailto:barbara.bohle@meduniwien.ac.at)

**ACKNOWLEDGEMENTS**

We thank Anja Drescher for helpful discussions regarding the interpretation of SPR results. This study was supported by the Austrian Science Fund (FWF), projects P32953 and I4437, the Austrian Jubiläumsfond, project ÖNB17947, and by the Danube Allergy Research Cluster, Country of Lower Austria, and Medical University of Vienna, Austria. The project was also supported by EQ-BOKU VIBT GmbH and the BOKU Core Facility Biomolecular & Cellular Analysis. The Austrian Federal Ministry for Digital and Economic Affairs, the National Foundation for Research, Technology and Development, and the Christian Doppler Research Association are gratefully acknowledged.

**MATERIAL AND METHODS**

**Characteristics of the allergic donor of peripheral blood mononuclear cells (PBMC)**

The individual received a daily sublingual dose of 25 µg of recombinant (r) Mal d 1 for 16 weeks. The sublingual dose of rMal d 1 which induced an oral allergy syndrome was 1.6 µg before and 10.97 µg after treatment, respectively. The serum before treatment contained 7.24 kU_A_/l of rMal d 1-specific IgE, 126.78 ng/ml of rMal d 1-specific IgG1, and 130 ng/ml of rMal d 1-specific IgG4 antibodies (Abs). After treatment 6.92 kU_A_/l of specific IgE, 398.4 ng/ml of IgG1, and 200 ng/ml of IgG4 Abs were detected. The post-treatment serum displayed 91.1% of blocking activity in basophil inhibition tests with apple extract and 95% with rMal d 1. This IgE-blocking activity dropped to 4.7% if the post-SLIT sample was depleted from IgG1 and to 73.7% if it was devoid of IgG4 Abs. The details of the respective analyses were published in (1, 2).

**Yeast library construction, selection and screening**

Total RNA was isolated from 1x10^7^ PBMC using TRIzol-based protocol (modified from (3)) and 500 ng were reverse transcribed with SuperScript™ IV First Strand Synthesis system (Thermo Fisher Scientific, Waltham, Massachusetts, USA). Amplicons of heavy and light chains were produced using a Human-Ig Primer Set (Merck, Darmstadt, Germany). Heavy and light chains were amplified with 5‘-primers corresponding to diverse germline sequences and 3‘-primers aligning to the end of the variable domain of the respective chain and incorporating the sequences overlapping with the pYD1-based yeast vectors required for gap-driven homologous recombination. Chemical transformation was used to create a light chain library from 9 sublibraries in a MATα *S. cerevisiae* strain BJ5464 (ATCC 208288). A heavy chain library was created from 5 sublibraries covering VH1, VH3, VH5, VH6, and VH7 in a MATa strain EBY100 (Thermo Fisher Scientific). Transformation of about 10 μg of VL-amplicon or VH-amplicon together with yeast vector resulted in libraries of 1.1x10^7^ and 1.6x10^7^ independent clones as determined by plating a dilution series to SD-minimal medium (1x drop-out supplements (Sigma Aldrich, Vienna, Austria), 3.4 mg/mL yeast-nitrogen base (Thermo Fisher Scientific), 10 mg/mL ammonium sulphate, 0.1 M potassium phosphate, pH 6.0, 2% glucose, 100 U/mL penicillin and 100 µg/mL streptomycin), supplemented with 80 µg/mL tryptophan or 100 µg/mL leucine, respectively. Sanger sequencing revealed a correctness of 96.7% and 95.1% of the clones in each library. Yeast mating and induction of the mated library of 1.38x10^8^ in size proceeded as described (4). 65% of the cells bound FITC-labelled anti-V5 Ab (Thermo Fisher Scientific, MA1-80281) confirming the presence of the Aga2p-anchored VH-CH1 fragment. 15.9% of the cells bound to FITC-labelled anti-human-kappa Ab (Sigma-Aldrich, F-3761) confirming the presence of a secreted light chain (Fig. S1A). rMal d 1 was biotinylated at a biotin:protein ratio of 5:1 with EZ-Link™ NHS-LC-LC-Biotin reagent and used for specific staining followed by streptavidin-AlexaFluor 647 (all from Thermo Fisher Scientific). To process a representative aliquot of the library, magnetic beads-activated cell selection (MACS) using SuperMACS™ II Separator and streptavidin magnetic beads (MACS Miltenyi, Bergisch Gladbach, Germany) with 4x10^9^ library cells was followed by two FACS-based selection rounds with Sony 8000SH sorter apparatus. Briefly, yeast cells were blocked with Candor blocking solution (CANDOR Bioscience GmbH, Wangen, Germany) for 30 min at room temperature, stained with biotinylated rMal d 1 for 1 h and streptavidin-AlexaFluor-647 for 30 min on ice. Complete light chains were detected by anti-kappa-FITC Ab and served for normalization of the expression level. The top 0.1% double positive cells were selected. In each sorting round at least the 5-fold output of the previous round was processed. Visible enrichment of individual rMal d 1-binding yeast clones was followed by their screening with 50 nM rMal d 1 and the kappa chain using Guava flow cytometer (Thermo Fisher Scientific). After one MACS selection and two FACS-sorting rounds, 0.5% of the population were positive (Fig. S1B). 17/24 sorted single clones were rMal d 1-positive, 16 of them had a correct Ab sequence, and 13 were unique. Multiple occurrences of the same heavy chain sequence were found. Altogether 3 different heavy chain complementarity determining regions (CDR)3-sequences were discovered. The yeast-displayed Fabs were reformatted to soluble IgG Abs for expression in mammalian cells: variable sequences were cloned into pTT5-based vectors (Canadian National Research Council, Ottawa, Ontario, Canada) using NEB Builder reagent (New England Biolabs, Ipswhich, MA, USA). After transformation into *E. coli* TOP10-electrocompetent cells (Thermo Fisher Scientific) the correct plasmids (25 μg) were transfected to HEK293-6E cells (1.5-2.0x10^6^/mL in 25 mL) in 1:1 mass ratio of both chains, combined with double mass of polyethylenimine reagent (Polysciences, Warrington, PA, USA). Cells were cultured in a humidified atmosphere with 5% CO_2_ at 37°C for 5 days, with feeding with TN-1 tryptone to a final concentration of 0.5% on day 2. Abs were isolated using Protein A chromatography as described (5). Protein concentration was determined using Nanodrop 2000C spectrophotometer (Thermo Fisher Scientific). About 20 μg of the purified Ab was subjected to size exclusion chromatography (SEC) on a Superdex 200 Increase 10/300 GL column (Cytiva, Uppsala, Sweden) connected to Shimadzu Prominence LC20 system in PBS/0.2M NaCl, run at 0.75 mL/min, with Bio-Rad gel filtration standard mixture for calibration.

**Light chain pool expansion**

The heavy chain of the diploid yeast clones was isolated using Zymoprep kit II (ZymoResearch, Irvine, CA, USA), amplified in *E. coli* TOP10 cells, purified using Nucleospin kit II (Macherey-Nagel, Düren, Germany), and re-transformed into the EBY100 strain. Each clone harboring a single heavy chain was re-mated with the complete BJ5464-cloned library of the light chains to a final size of 7.2x10^7^ for K1.1 and 1.2x10^8^ for K2.4. Phenotypic quality control was performed as described above. Parental yeast clones were stained with a dilution series of rMal d 1 to determine the threshold antigen concentration for selections. Affinity maturation libraries were processed as described above, but the rMal d 1 concentration used for staining was set to 1 nM for the K1-1 and 2 nM for the K2-4 library. After enrichment of rMal d 1-binding cells, individual clones were plated onto solid media and screened with the same antigen concentration.

**Characterization of human monoclonal antibodies (mAbs)**

For ELISA, biotinylated rMal d 1 (2.5 μg/mL) was coated to NUNC streptavidin-coated Immobilizer plates. After saturation with 4% bovine serum albumin (BSA, Sigma-Aldrich) for 1 h, Abs were incubated in a 1:3 dilution series starting from 666 nM concentration in 2% BSA-PBS for 1 h. Binding was detected with anti-human-kappa-horseradish peroxidase (HRP) conjugate (Sigma-Aldrich A-7164) added in 2% BSA-PBS for 45 min. The reaction of 5‘,5‘,3‘,3‘-tetramethylbenzidine was stopped with an equal volume of 30% H_2_SO_4_ and the absorbance at 450/620 nm was recorded using a Tecan Sunrise reader (Tecan, Männedorf, Switzerland). EC_50_ values were evaluated with GraphPad Prism 5.0 program (GraphPad Software Inc., San Diego, California). The murine IgG1 mAb BIP1 served as positive control and was detected with anti-mouse-Fc-HRP conjugate (Sigma-Aldrich A-2554).

Surface plasmon resonance (SPR) was performed using a Biacore™ T200 instrument (Cytiva) at 25° C. First, an anti-human IgG Ab was coupled to an immobilization level of around 7000 RU in flow cells 1 and 2 of a Series S CM5 sensor chip using the Human Antibody Capture kit (all from Cytiva). Abs were diluted in running buffer HBS-P+ (10 mM HEPES, 150 mM NaCl, 0.05 % Surfactant P20; Cytiva) and injected in flow cell 2 to reach a binding level of 185-350 RU, aiming for an R_max_ of 45-80 RU. rMal d 1, diluted in HBS-P+, was injected at a flow rate of 30 µL/min over both flow cells at concentrations ranging from 625-4.88 nM for 240 s in a multicycle kinetic assay setup. Dissociation was monitored for 720 s. HBS-P+ injections served as blank runs. The chip surface was regenerated by a 30 s pulse of 3 M MgCl_2_ at a flow rate of 20 µL/min. The kinetic evaluation was performed in Biacore™ T200 Evaluation Software (Cytiva) using referenced and blank subtracted sensorgrams. Curves were fitted using the 1:1 Langmuir binding model.

**Inhibition of basophil activation**

Eight different untreated individuals (7 male, 1 female, 22-63 years) with birch pollen-related apple allergy as documented by hayfever in spring, reported oral allergy syndrome to fresh apple, and Bet v 1- and Mal d 1-specific IgE levels of >0.35 kU_A_/ml (ImmunoCAP, Thermo Fisher Scientific), were included after informed consent and ethical clearance by the local ethics committee (EK1344/2018). rMal d 1 and mAbs were diluted in HEPES calcium buffer (HCB, pH 7.4) supplemented with BSA (1 mg/mL) and IL‑3 (2 ng/mL). The murine IgG1 mAbs BIP1 and BIP3 served as positive and negative controls, respectively. In each experiment, six rMal d 1 concentrations (10 µL) were incubated with either HCB (10 µl) or mAb (10 µL) at indicated molar ratios for 1 h at room temperature. Then, heparinized blood was added for 15 min at 37°C. After addition of HEPES/EDTA buffer (pH 7.4) cells were stained with anti-CD123 (PerCP), anti-CCR3 (APC), and anti-CD63 (PE) Abs (BioLegend, San Diego, California) and acquired on a FACSCanto II using FACSDiva Software Version 6.1.3 (BD Biosciences, San Jose, California) after lysis of erythrocytes. CD63 expression was assessed on CD123^+^CCR3^+^ cells. For each donor, all assays with rMal d 1 concentrations inducing 18-69% CD63^+^ basophils were normalized to 100% activation and the percentage of inhibition of activation by mAbs was calculated. Then, the mean value of inhibition for each mAb was calculated per donor.

**Statistical analysis**

Statistical analyses were performed in GraphPad Prism 9.3.1 (GraphPad Software Inc.) using the Friedman test followed by Dunn’s multiple comparisons test. Differences were considered significant when p ≤ 0.05.

**References**

1. Kinaciyan T, Nagl B, Faustmann S, Frommlet F, Kopp S, Wolkersdorfer M, et al. Efficacy and safety of 4 months of sublingual immunotherapy with recombinant Mal d 1 and Bet v 1 in patients with birch pollen-related apple allergy. *J Allergy Clin Immunol* 2018;**141**(3):1002-1008.

2. Sanchez Acosta G, Kinaciyan T, Kitzmuller C, Mobs C, Pfutzner W, Bohle B. IgE-blocking antibodies following SLIT with recombinant Mal d 1 accord with improved apple allergy. *J Allergy Clin Immunol* 2020;**146**(4):894-900 e892.

3. Chomczynski P, Sacchi N. Single-step method of RNA isolation by acid guanidinium thiocyanate-phenol-chloroform extraction. *Anal Biochem* 1987;**162**(1):156-159.

4. Sadio F, Stadlmayr G, Eibensteiner K, Stadlbauer K, Ruker F, Wozniak-Knopp G. Methods for Construction of Yeast Display Libraries of Four-Domain T-Cell Receptors. *Methods Mol Biol* 2020;**2070**:223-248.

5. Benedetti F, Stadlbauer K, Stadlmayr G, Ruker F, Wozniak-Knopp G. A Tetravalent Biparatopic Antibody Causes Strong HER2 Internalization and Inhibits Cellular Proliferation. *Life (Basel)* 2021;**11**(11).

**RESULTS**

**Figure S1. Yeast library characterization and antigen affinity selection**


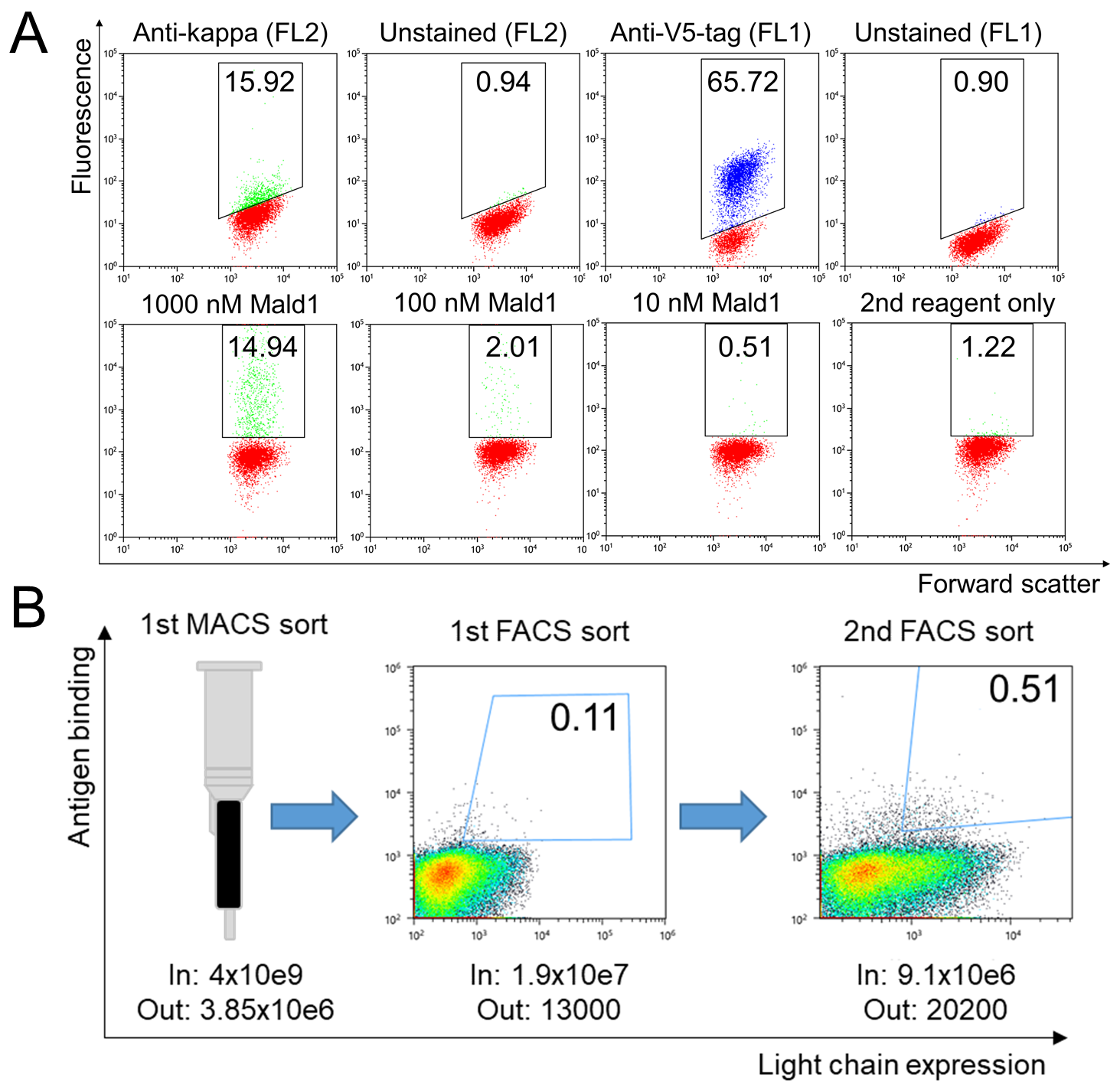


A, Percentage of positive cells after staining with anti-kappa or anti-V5 Abs, indicating the display of light or heavy chain (upper panel) or graded antigen concentrations (lower panel);

B, Sorting procedure with percentages of positive cells and the numbers of processed and selected cells.

**Figure S2. Characterization of Mal d 1-specific mAb.** A, Size exclusion purification protocols (MWS: molecular weight standard); B, Binding to biotinylated-rMal d 1 assessed by ELISA.


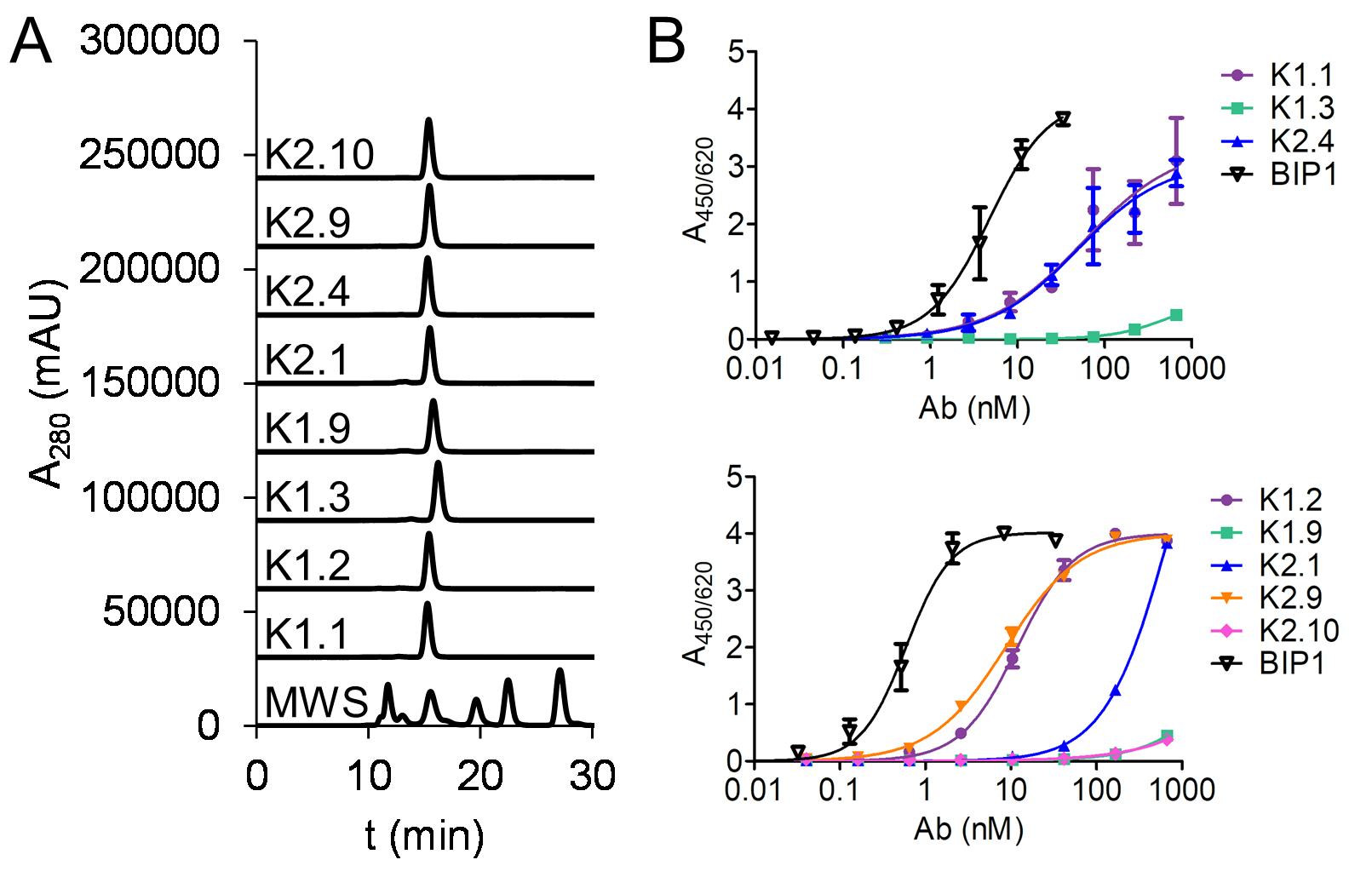


**Figure S3. Binding kinetics and affinity of mAbs.** mAb were captured on a CM5 chip with covalently immobilized anti‑human IgG. Mal d 1 was injected in a multicycle assay format in concentrations ranging from 625–19.5 nM (K1.1 and K2.4) or 312.5-4.9 (all others). The interactions were evaluated after reference and blank subtraction using the 1:1 Langmuir binding model.


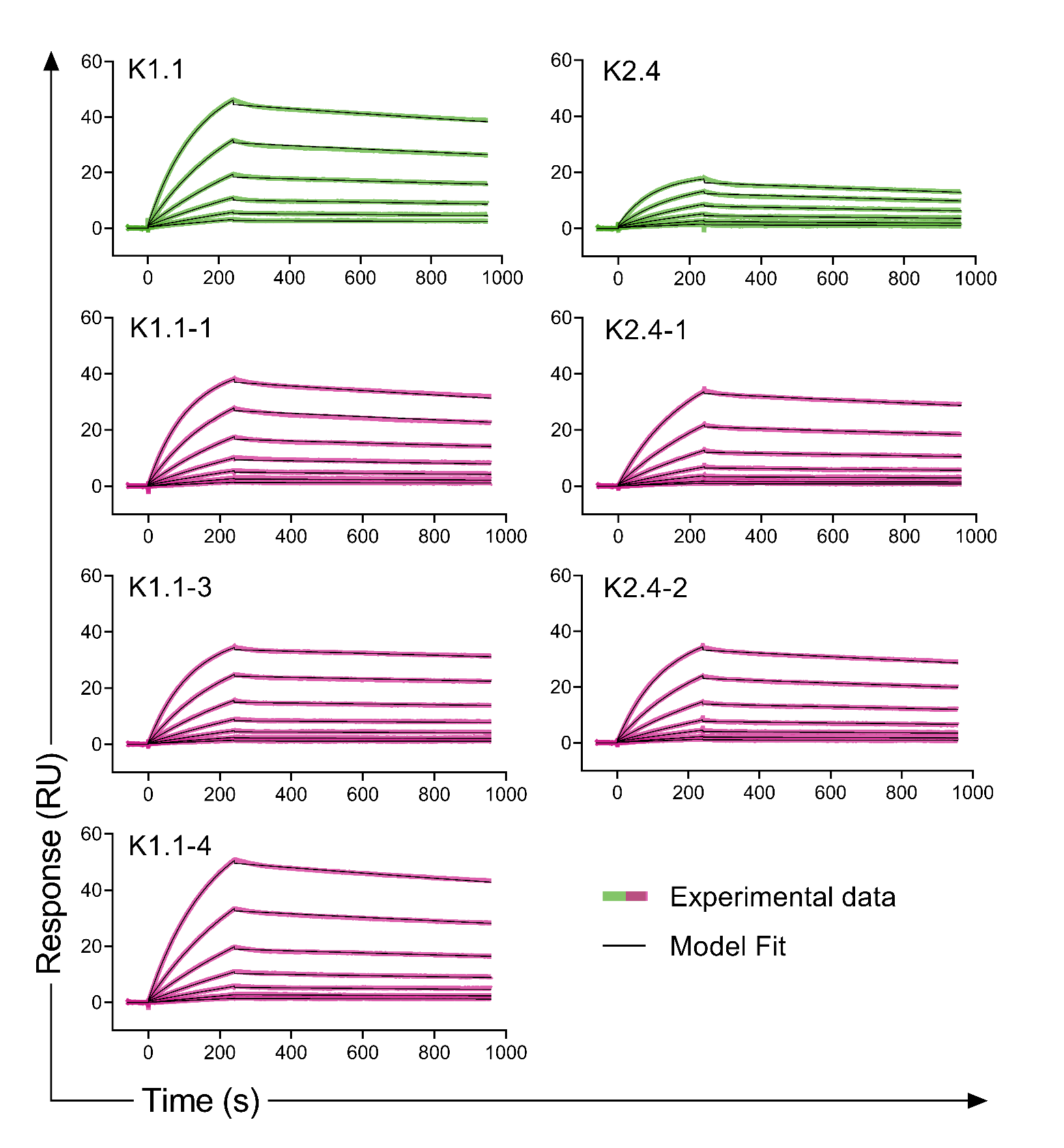

Supplement: Supplementary file 1 — Appendix S1. [file ALL-78-2543-s001.docx]
